# Role of individual dispersal in genetic resilience in fluctuating populations of the gray‐sided vole *Myodes rufocanus*

**DOI:** 10.1002/ece3.7300

**Published:** 2021-02-21

**Authors:** Yasuyuki Ishibashi, Kenichi Takahashi

**Affiliations:** ^1^ Hokkaido Research Center Forestry and Forest Products Research Institute Sapporo Japan; ^2^ Hokkaido Institute of Public Health Sapporo Japan; ^3^ Present address: Hokkaido Pest Control Association Sapporo Japan

**Keywords:** arvicoline, cluster compositions, density fluctuation, dispersal, genetic diversity

## Abstract

Population densities of the gray‐sided vole *Myodes rufocanus* fluctuate greatly within and across years in Japan. Here, to investigate the role of individual dispersal in maintaining population genetic diversity, we examined how genetic diversity varied during fluctuations in density by analyzing eight microsatellite loci in voles sampled three times per year for 5 years, using two fixed trapping grids (approximately 0.5 ha each). At each trapping session, all captured voles at each trapping grid were removed. The STRUCTURE program was used to analyze serially collected samples to examine how population crashes were related to temporal variability, based on local‐scale genetic compositions in each population. In total, 461 and 527 voles were captured at each trapping grid during this study. The number of voles captured during each trapping session (i.e., vole density) varied considerably at both grids. Although patterns in fluctuations were not synchronized between grids, the peak densities were similar. At both grids, the mean allele number recorded at each trapping session was strongly, positively, and nonlinearly correlated with density. STRUCTURE analyses revealed that the proportions of cluster compositions among individuals at each grid differed markedly before and after the crash phase, implying the long‐distance dispersal of voles from remote areas at periods of low density. The present results suggest that, in gray‐sided vole populations, genetic diversity varies with density largely at the local scale; in contrast, genetic variation in a metapopulation is well‐preserved at the regional scale due to the density‐dependent dispersal behaviors of individuals. By influencing the dispersal patterns of individuals, fluctuations in density affect metapopulation structure spatially and temporally, while the levels of genetic diversity are preserved in a metapopulation.

## Graphical abstract

Allelic diversity was strongly, positively, and non‐linearly correlated with density at two trapping grids, although large fluctuations in density were observed. At each grid, if the density was similar, the mean number of alleles observed was also similar, irrespective of trapping sessions.

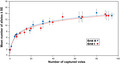

## 1 INTRODUCTION

Fluctuations in population size have been recorded in arvicoline rodents, which include voles and lemmings (Elton, 1942; Krebs et al., 1969; Taitt & Krebs, 1985), although the causes of these fluctuations have not been fully clarified (Oli, 2019). Arvicoline populations often undergo 3–5‐year cycles of 10‐ to 100‐fold changes in density (Stenseth, 1999). During a period of fluctuation, after reaching peak density, populations are almost exterminated, or crash, within a few months. However, true extinction does not occur because those populations recover to reach peak density again in several years. With such fluctuations, population genetic diversity may decrease due to repeated bottlenecks (Frankham et al., 2017). However, high levels of population genetic diversity are frequently reported (e.g., Berthier et al., 2005, 2006; Ehrich et al., 2009; García‐Navas et al., 2015; Gauffre et al., 2014; Pilot et al., 2010). In their review of northern population cycles, Norén and Angerbjörn (2014) outlined nine predictions covering the direct and genetic feedback consequences of population cycles on genetic variation and population structure and verified them with empirical evidence. They concluded that although genetic variation in northern cyclic populations is generally high and the geographic distribution and amount of diversity are usually suggested to be determined by various forms of context‐ and density‐dependent dispersal exceeding the impact of genetic drift, the signatures of microevolutionary processes such as genetic drift and selection are weaker and obscured by density‐dependent dispersal. In fluctuating vole populations, it is thought that genetic variation is maintained through a combination of ecological and evolutionary processes. These include (1) differences in dispersal patterns throughout fluctuations, (2) crash phases with a short duration that are accompanied by weak genetic drift, and (3) the rapid accumulation of new alleles through mutation or immigration (Gauffre et al., 2014; Norén & Angerbjörn, 2014). Berthier et al. (2005) suggested that in the fossorial water vole *Arvicola terrestris*, dispersal rates are low among isolated remaining populations during periods of low density, resulting in substantial differentiation even among nearby patches. Subsequently, as the populations increase to peak‐density levels, the populations expand spatially, facilitating the exchange of individuals between neighboring populations; thus, populations become more similar. Additionally, Berthier et al. (2006) showed that genetic drift, which has a strong influence during periods of low density, and migration, which mainly occurs as population size increases, interact closely to maintain high genetic diversity within a metapopulation (i.e., a population of populations) (Hanski & Gilpin, 1997) at the regional scale (also see Gauffre et al., 2014). Although there is clarity regarding the mechanism by which genetic variation is preserved within a metapopulation, there still is a lack of information regarding how genetic diversity varies with density at the local scale, and how this process is influenced by density‐dependent dispersal.

Before and after a population crash, there may be substantial changes in the genetic compositions of individuals that inhabit a specific area. Considering the mechanism by which genetic variation is maintained, and because of breeding among new immigrants in populations with few or no remaining previous residents, local populations that have recovered from a crash would likely acquire different combinations of alleles across loci, compared to a precrash population. Recently, Bayesian inference analyses have been performed in many studies to spatially delineate populations (e.g., Ouchene‐Khelifi et al., 2018; Pelletier et al., 2011; Priadka et al., 2019; Viengkone et al., 2016; Wang et al., 2017). Bayesian inference analysis of samples that are collected serially at a fixed site will be likely to show drastic changes in the cluster compositions of individuals, reflecting density crashes.

**FIGURE 1:**
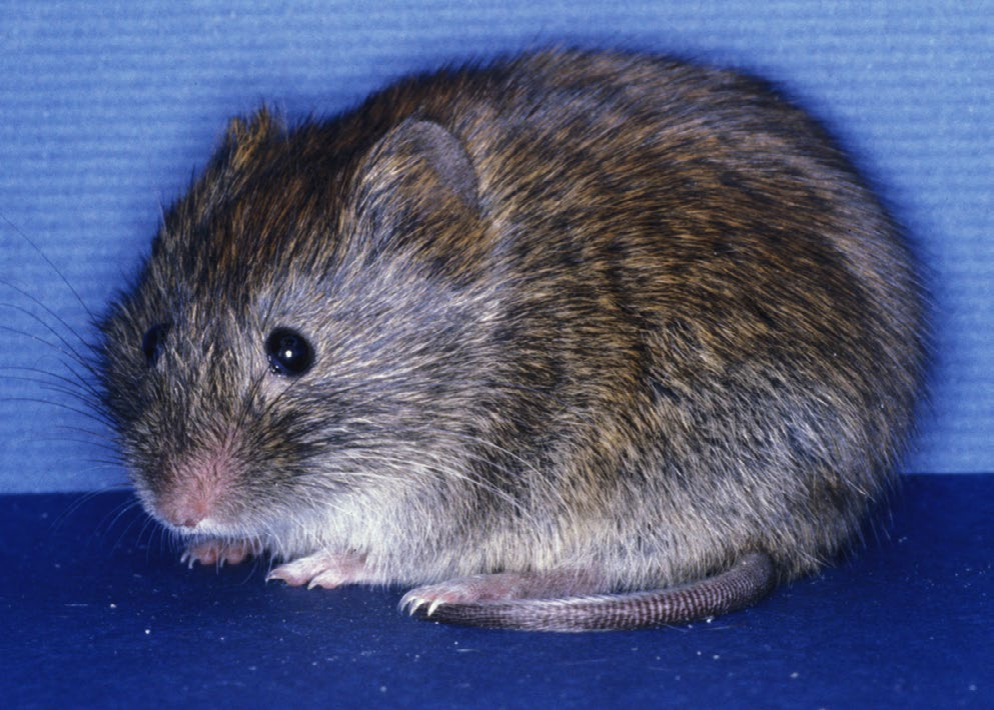
The gray‐sided vole *Myodes rufocanus*. In Japan, the vole occurs only on Hokkaido, the northernmost island of Japan

Populations of the gray‐sided vole *Myodes rufocanus* (Figure 1) have fluctuated greatly within and across years in Hokkaido, the northernmost island of Japan (Ohdachi et al., 2015; Saitoh, 1987; Saitoh et al., 1998). This small rodent (~50 g) has a short life‐span (mean, 193 days for bred females; Saitoh, 1990). During the breeding season, the reported home‐range lengths are ~40 and 65 m for females and males, respectively (Ishibashi & Saitoh, 2008a). Individuals of both sexes disperse from their natal sites when they reach sexual maturity (Ishibashi & Saitoh, 2008b; Saitoh, 1995). Male‐biased dispersal is well‐known. Within a 3‐ha enclosure, approximately 70% of females reproduced within two home‐range lengths of their natal sites, whereas more than 80% of the males bred more than 33 m away from their natal sites within their birth year, with a median distance of 103.5 m (interquartile range, 56.9–135.0 m) recorded (Ishibashi & Saitoh, 2008b). During the non‐breeding season (i.e., in winter), voles had smaller ranges than during the breeding season; most seemed to nest communally (Ishibashi et al., 1998; Saitoh, 1989). Vole abundance usually reaches a maximum at the end of the autumn breeding season. Peak densities in areas with the highest vole abundances can reach 100–200 or more individuals per hectare (Saitoh et al., 1998). A time‐series analysis of long‐term census data in Hokkaido grouped local populations into four types based on their fluctuation patterns, with the most common type showing high fluctuation among years, a ratio of maximum to minimum density of >10, and peaks at intervals of 3–4 years (Saitoh, 1987). Cyclicity is obvious among populations located in northeastern Hokkaido (Saitoh et al., 1998). In this study, we examined the variations in microsatellite loci diversity in voles three times per year for 5 years at two fixed sites, from which all captured voles were removed in each trapping session, to address the role of density‐dependent dispersal in the maintenance of genetic diversity within fluctuating vole populations. We also used a Bayesian inference application to analyze serially collected samples, to explore the relationship between population crashes and local‐scale temporal variability in the genetic composition of each population.

## 2 MATERIALS AND METHODS

### 2.1 Study site and sampling protocol

**FIGURE 2:**
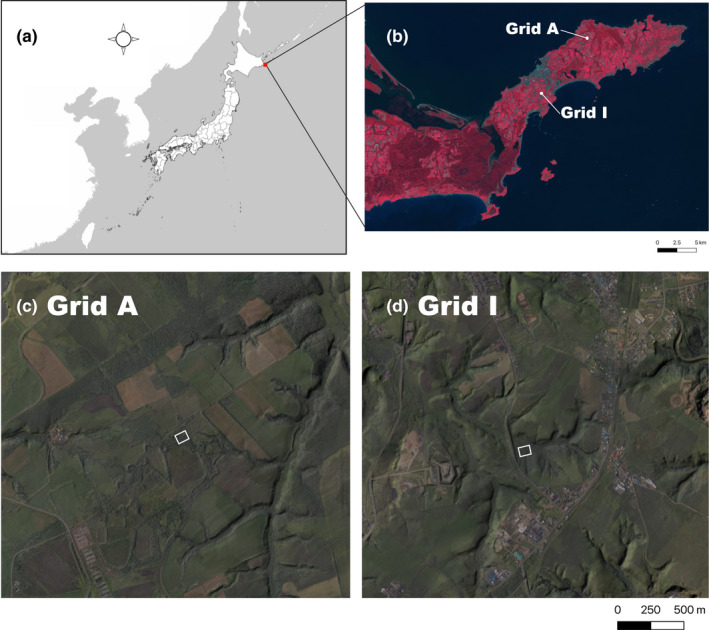
(a) Location of Nemuro Peninsula, Hokkaido, Japan. (b) Locations of two study sites. Areas with some vegetation are colored red. This false‐color composite image was created with QGIS 3.14.0‐Pi (QGIS.org, 2020) using Landsat‐8 images. (c, d) Composite images of aerial photographs and shaded‐relief maps for grids A and I, which were constructed using QGIS. Squares indicate the positions of each trapping grid (0.5 ha per grid). Aerial photographs and shaded‐relief maps were obtained from the Geospatial Information Authority of Japan

On the Nemuro Peninsula, the breeding season of the gray‐sided vole is from mid‐April to early October. To accurately identify changes in population density and collect liver samples during the breeding season, voles were captured three times per year (mid‐spring/late May, mid‐summer/early August, and late autumn/early October) for 5 years (2002–2006) at two fixed sites on Nemuro Peninsula—grids A and I (located 9.3 km apart; Figure 2). Grid A was located in a shelterbelt and grid I was located in a low‐lying and damp area. To investigate parasitic *Echinococcus multilocularis* in the liver, one of the authors (KT) has collected voles three times per year at these grids since the late 1980s with the permission of the Hokkaido Government. Tissue samples were collected from these voles during 2002–2006 for DNA extraction. During each trapping session, 50 Sherman‐type traps were arranged in a 5 × 10 grid pattern at an interval of 10 m (~0.5 ha) with a handful of oatmeal in each trap for three nights (i.e., a total of 150 trap‐nights per session). Under this sampling protocol, almost all weaned gray‐sided voles in the grid would be captured, allowing us to estimate vole density per 0.5 ha at the study sites. Upon capture, voles were euthanized by cervical spine dislocation. At the laboratory of the Nemuro Subprefectural Bureau of the Hokkaido Government, a few small pieces of liver per vole were fixed in 99.5% ethanol after biopsy. Liver samples were stored at −80°C in Sapporo, Hokkaido until DNA extraction. To determine whether similar density fluctuation occurs around the grids, we concurrently performed additional trapping in the areas surrounding grids A and I, during which voles were captured using 49 or 50 live traps on three nights; these voles were released shortly after capture.

Nemuro Peninsula has a subarctic climate (Saito & Okitsu, 1987). Because of a cold sea current (i.e., the Kuril Current), the eastern part of Hokkaido is often enveloped in sea fog from early summer to early autumn. The peninsula has been impacted by anthropogenic activities since the late 19th century, with the creation of many pastures. Dwarf bamboo *Sasa nipponica*, the main habitat of gray‐sided voles in eastern Hokkaido, is distributed in various places, such as around pastures, along streams, and in shelterbelts with deciduous shrubs (e.g., *Alnus hirsuta*) as undergrowth. Although the middle of the peninsula has been urbanized, most of the peninsula remains vegetated (Figure 2b). Graminivorous gray‐sided voles can inhabit any vegetated areas; therefore, our trapping in 0.5‐ha grids might not have affected the population density at the study sites very much due to the small area of the trapping grids, compared with the large extent of the surrounding vegetation (Figure 2c,d).

### 2.2 Genotyping

Genomic DNA was extracted from each liver sample using the conventional phenol‐chloroform method (Sambrook et al., 1989). Polymerase chain reaction (PCR) amplification was performed at eight microsatellite loci. To avoid contamination of post‐PCR products, eight‐strip tubes were used and always centrifuged before the caps were opened. Six loci (i.e., MSCRB‐01, ‐04, ‐06, ‐07, ‐11, and ‐13) were amplified as reported previously (Ishibashi & Saitoh, 2008a,b; Ishibashi et al. 1995, 1997) with an ABI 2720 Thermal Cycler system (Applied Biosystems) using a 10‐µl reaction mixture containing 0.25 µM of each primer, 0.2 mM dNTPs, 10 mM Tris‐HCl (pH 8.3), 50 mM KCl, 1.5 mM MgCl_2_, 0.001% (w/v) gelatin, 0.25 U AmpliTaq Gold DNA polymerase (Applied Biosystems), and approximately 100 ng genomic DNA. Two other loci, MSCRB‐09 and −10 (GenBank accession nos. AB248757 and AB248758), were amplified using the same reaction mixture mentioned above, with the primer sets 5′‐TAGGA ACAGC TGGAG AACCA C‐3′ and 5′‐TGCAC ACTCT AAATA CCATG TGCA‐3′ for MSCRB‐09 and 5′‐TGCCC CAATC TGTCT TCCAC‐3′ and 5′‐TCAGA CTCAG TACAG TGAAT CT‐3′ for MSCRB‐10. Both amplification processes used an annealing temperature of 54°C. The PCR process was as follows: incubation for 10 min at 95°C, 35–55 cycles of 20 s at 93°C, 15 s at a locus‐specific annealing temperature, and 20 s at 72°C. Genotyping was performed with an ABI PRISM 310 Genetic Analyzer (Applied Biosystems) using GeneScan Analysis 3.1.2 and Genotyper 2.5 software (Applied Biosystems).

### 2.3 Genetic characteristics

Basic information such as the number of observed alleles and heterozygosity level was calculated using GenAlEx 6.51b2 (Peakall & Smouse, 2006, 2012). Levels of genetic differentiation among trapping sessions (*F*_ST_) were also calculated using GenAlEx; data were used from trapping sessions in which five or more voles were captured. Allelic richness, a measure of the number of alleles within samples that considers the differences in sample size (Mousadik & Petit, 1996), was calculated using the *hierfstat* package (Goudet, 2005) in R 3.6.2 software (R Development Core Team, 2019). To ensure that the heterozygosity of all individuals was measured on an identical scale, we standardized individual heterozygosity by calculating the proportion of heterozygous typed loci relative to the mean heterozygosity of typed loci (Coltman et al., 1999). Using the web‐based version of Genepop (Raymond & Rousset, 1995; Rousset, 2008; http://genepop.curtin.edu.au/), the presence of Hardy–Weinberg equilibrium was tested at each locus for each trapping session; linkage disequilibrium among loci was tested using samples from trapping sessions in which null alleles were likely to be absent. For each session, the presence of null alleles was assessed at each locus using the program MICRO‐CHECKER 2.2 (Van Oosterhout et al., 2004).

### 2.4 Temporal changes in cluster composition

To investigate temporal changes in cluster composition among individuals, Bayesian clustering analysis of serially collected samples was performed using STRUCTURE 2.3.4 (Falush et al., 2003; Hubisz et al., 2009; Pritchard et al., 2000), which was also used to estimate the population membership of each individual. The proportions of clusters of admixed individuals were estimated using the admixture model. The models used in this program version could detect structure at lower levels of divergence, compared to the original models, with the inclusion of information regarding sampling locations (Hubisz et al., 2009). Because the presence of null alleles may affect analytical outcomes, all loci suspected to have null alleles were excluded from our analyses. The number of clusters (*K*) varied from one to eight. Each run, replicated 100 times, comprised a burn‐in period of 10^4^ and 2 × 10^4^ iterations with an admixture model under default settings, with the exception of option LOCPRIOR, in which the order of trapping sessions was considered to represent sampling location. Using the R package *pophelper* 2.3.0 (Francis, 2017), the most probable number of clusters was inferred from Δ*K* plots (Evanno et al., 2005); plots of membership coefficients (*q* values) were drawn for the inferred *K* value, for which individual assignments to clusters were determined using the CLUMPP 1.1.2 program (Jakobsson & Rosenberg, 2007).

## 3 RESULTS

### 3.1 Fluctuations in density

**FIGURE 3:**
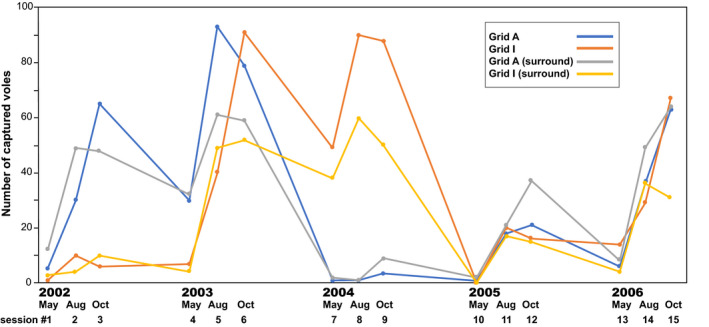
Fluctuations in gray‐sided vole density during the study period (May 2002–October 2006) in two 0.5‐ha grids (A and I) and their surroundings. Each point indicates the number of voles captured. See the text for trapping methodology. Numbers at the bottom of the figure are trapping session numbers

The number of voles captured at each session (i.e., vole density) varied considerably at both grids, despite constant trapping efforts (Figure 3). We captured 461 and 527 total voles at grids A and I, respectively. Because of our trapping scheme (i.e., all captured voles were removed at each trapping session), the captured voles consisted of immigrants into the grids from surrounding areas and their offspring. Although patterns in fluctuation were not synchronized between grids, the peak densities were similar. At grid A, vole density increased through 2002 and reached high levels in 2003. Then, the vole population crashed, such that low densities were observed until May 2005 (sessions 7–10), during which five voles were captured. Subsequently, vole density continued to increase (sessions 11–15). At grid I, only one vole was captured in May 2002 (session 1). Because one of the authors (KT) observed a peak in density in the previous year (October 2001), we were able to identify a crash in the spring of 2002. Densities were high the next 2 years, in 2003 and 2004; the population then crashed again, such that no voles were captured in the spring of 2005 (session 10). Vole density increased again after the summer of 2005 (sessions 11–15). The patterns of fluctuation observed during the additional trapping sessions in the areas surrounding the grids were similar to those observed at the study's trapping grids (Figure 3).

### 3.2 Genetic diversity

**FIGURE 4:**
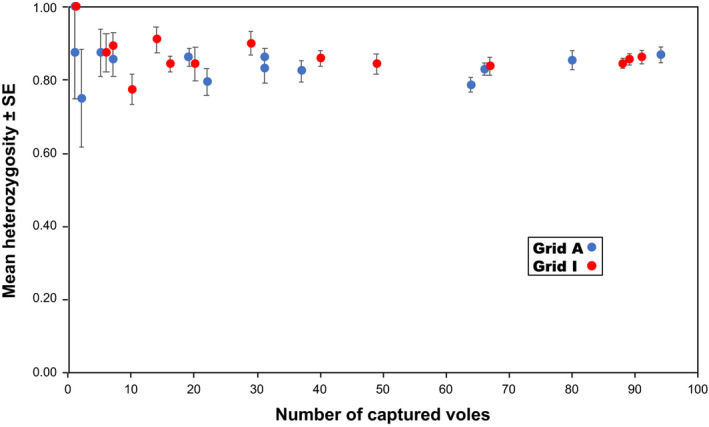
Relationship between density and observed heterozygosity. Mean heterozygosity levels among eight microsatellite loci were calculated. Vertical bars indicate standard errors (*SE*s). No correlations were observed in either grid (Spearman's rank correlation test, *p* > .089 for both grids). Null alleles were not considered

**FIGURE 5:**
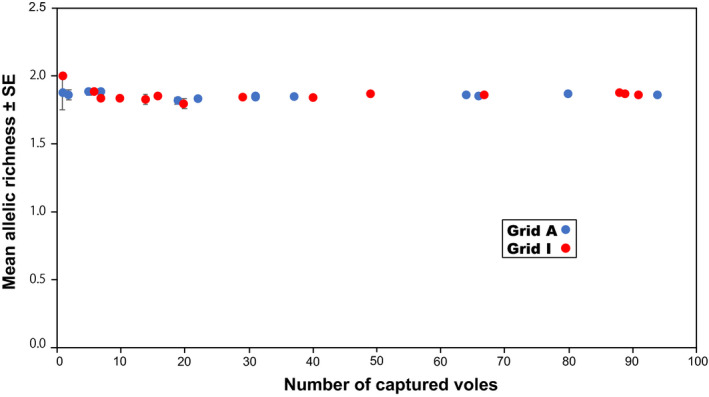
Relationship between density and allelic richness, which is a measure of the number of alleles within samples that considers the differences in sample size (Mousadik & Petit, 1996). Mean allelic richness levels were calculated for eight microsatellite loci. Vertical bars indicate *SE*s. No correlations were observed in either grid (Spearman's rank correlation test, *p* > .10 for both grids). Null alleles were not considered

**FIGURE 6:**
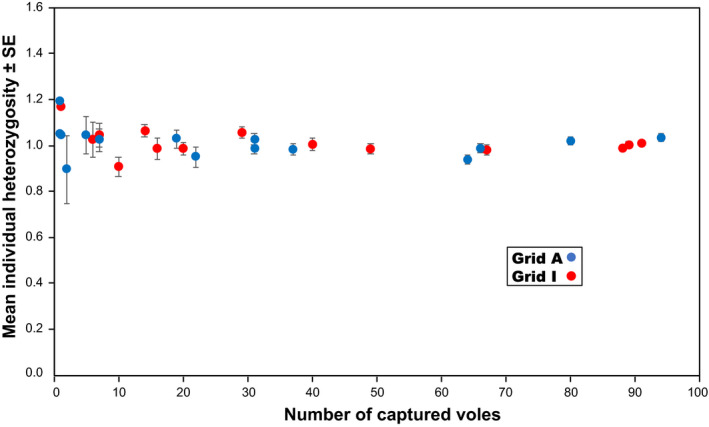
Relationship between density and standardized individual heterozygosity. Mean individual heterozygosity levels were calculated among individuals. Vertical bars indicate *SE*s. No correlations were observed in either grid (Spearman's rank correlation test, *p* > .089 for both grids). Null alleles were not considered

Genotypes were determined based on the eight microsatellite loci for all collected samples (461 and 527 at grids A and I), with the exception of a single animal, which had a missing value for one locus, MSCRB‐11 (Table 1; Appendices S1 and S2). At each grid, each locus examined exhibited high variability; allelic diversity was similar between grids. Each locus had 11 or more alleles. The locus MSCRB‐06 exhibited the greatest variability with 27 alleles across both sites. Throughout the study, levels of observed heterozygosity were high at both grids and similar between grids, with total mean ± standard error (*SE*) values of 0.850 ± 0.016 and 0.868 ± 0.009 for grids A and I, respectively. No correlation was observed between density and heterozygosity averaged among the eight loci at either grid (Figure 4). Similarly, no correlations were observed between density and the mean allelic richness or between density and the mean standardized individual heterozygosity at either grid (Figures 5 and 6); across all sessions, the mean allelic richness was 1.867 ± 0.012 and 1.860 ± 0.006 for grids A and I, respectively, and the mean standardized individual heterozygosity was 1.000 ± 0.007 and 1.000 ± 0.007 for grids A and I, respectively (Appendix S3). Moderate genetic differentiation was observed among trapping sessions at both grids, with mean *F*_ST_ ± *SE* values of 0.032 ± 0.002 and 0.046 ± 0.003 calculated for grids A (*N* = 11, *p* = .002) and I (*N* = 13, *p* = .001), respectively. Statistically significant departures from Hardy–Weinberg equilibrium were observed in three trapping sessions at grid A and four sessions at grid I. For each trapping session, significant departures from random union of gametes were observed at fewer than three loci at grid A and one locus at grid I (Bonferroni correction, *p* < .05/8; Appendices S1 and S2). In the tests of linkage disequilibrium among examined loci, some locus combinations exhibited significant nonrandom associations (Appendix S4). At grid A, an analysis of two trapping sessions excluding possible null alleles (sessions 3 and 5) revealed significant differentiation in six locus combinations (Bonferroni correction, *p* < .05/28). At grid I, an analysis of two trapping sessions (sessions 6 and 9), excluding possible null alleles, revealed significant differentiation in five locus combinations. However, of the locus combinations with significant nonrandom associations, no combinations were repeated across the sessions analyzed at each grid.

**TABLE 1:** Characteristics of microsatellite DNA loci in voles collected at grids A and I from 2002 to 2006

Locus	Grid A				Grid I			
	Individuals typed	Frequency of heterozygotes	An	Na	Individuals typed	Frequency of heterozygotes	An	Na
MSCRB‐01	461	0.831	13	6.933 ± 0.892	527	0.816	11	7.571 ± 0.661
MSCRB‐04	461	0.848	12	6.867 ± 0.856	527	0.871	14	8.714 ± 0.759
MSCRB‐06	461	0.829	27^a^	11.667 ± 1.851	527	0.890	27^a^	14.500 ± 1.747
MSCRB‐07	461	0.796	18^a^	8.200 ± 1.235	527	0.829	17	8.929 ± 0.855
MSCRB‐09	461	0.824	14^a^	7.800 ± 1.020	527	0.898	16	10.00 ± 0.845
MSCRB‐10	461	0.872	19^a^	9.800 ± 1.448	527	0.831	18	9.714 ± 1.008
MSCRB‐11	461	0.889	17^a^	8.800 ± 1.212	526^b^	0.867	15^a^	9.429 ± 0.936
MSCRB‐13	461	0.820	13	7.200 ± 0.890	527	0.837	14	7.786 ± 0.833

Note

In total, 461 and 527 voles from grids A and I, respectively, were examined. The number of individuals typed at each locus and frequency of heterozygotes are shown. An, total number of alleles observed during this study; Na, mean number of different alleles across sessions (± standard error).

^a^ Suspected null alleles identified using MICRO‐CHECKER (Van Oosterhout et al., 2004) were not included in this number.

^b^ One individual had a missing value.

The results of MICRO‐CHECKER analysis suggested that null alleles were present at MSCRB‐06, ‐07, ‐09, ‐10, and ‐11 for samples collected at grid A and at MSCRB‐06 and ‐11 for samples collected at grid I (Table 1; Appendices S1 and S2); however, the presence of null alleles was not suspected in some trapping sessions for these loci. Null alleles were suspected to be present in sessions 2 (MSCRB‐06), 4 (MSCRB‐07), 6 (MSCRB‐09), 12 (MSCRB‐06 and ‐10), 14 (MSCRB‐06), and 15 (MSCRB‐06 and ‐11) for grid A and in sessions 7 (MSCRB‐06), 8 (MSCRB‐11), and 15 (MSCRB‐06) for grid I.

### 3.3 Relationship between density and allelic diversity

**FIGURE 7:**
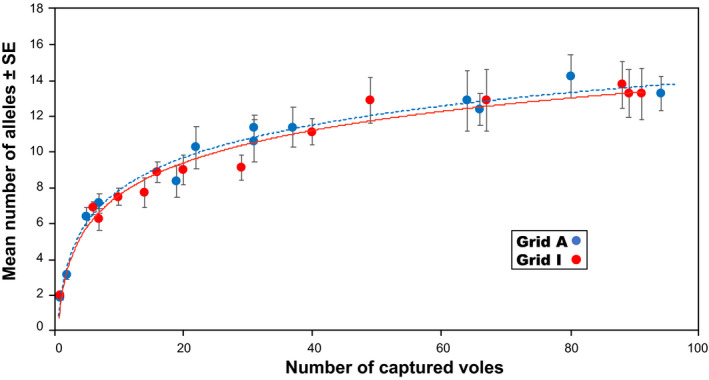
Relationship between density and allelic diversity. Allelic diversity is represented by the average number of different alleles observed among eight microsatellite loci, after increasing the number of alleles at loci suspected to have null alleles by one for each trapping session. Vertical bars indicate *SE*s. Dotted and solid lines represent model outcomes fitted to the means for grids A and I, respectively (see text)

As density increased, the average number of alleles observed at the eight loci increased, after the number of alleles at loci suspected to have null alleles had been increased by one for each trapping session (Figure 7). However, as vole density approached peak levels, the rate of increase gradually slowed. At both grids, we did not record all alleles for a locus in a single sample, even at peak density, except in one instance—all 11 alleles of MSCRB‐01 were recorded in session 9 (October 2004) at grid I.

To describe the nonlinear relationship between density and allelic diversity, the *nls* command in R was applied to obtain nonlinear least‐squares estimates of parameters for three assumed models after Crawley (2015) and Logan (2010) (Appendix S5). The appropriateness of the models was then assessed by comparing their Akaike Information Criterion (AIC) values (Burnham & Anderson, 2002). Although the coefficients of determination (*R* ^2^) were higher than 94% for all models, one model, *Y* = *a* + *b* × ln (*X*), where *Y* is the mean number of observed alleles and *X* is the density, produced both the highest *R* ^2^ values and lowest AIC values for both trapping grids (grid A: *a* = 1.81392, *b* = 2.62684, *R* ^2^ = 98.7%, AIC = 27.130; grid I: *a* = 1.5599, *b* = 2.6154, *R* ^2^ = 97.0%, AIC = 30.174; Figure 7).

**FIGURE 8:**
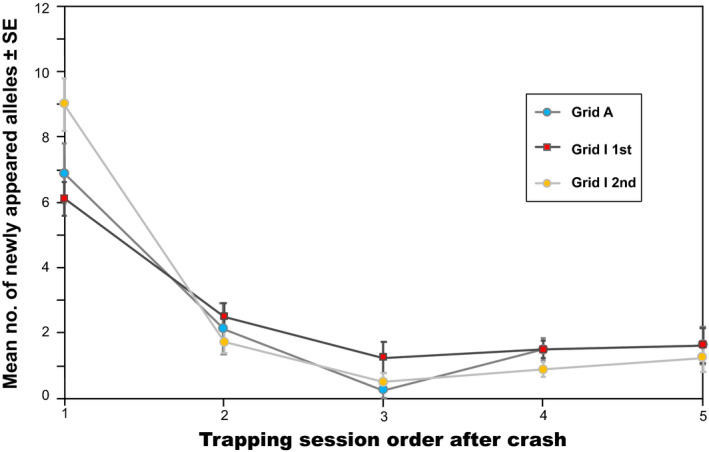
Numbers of newly appearing alleles after each crash. Alleles newly appearing in the first five trapping sessions after a crash were counted; alleles were counted only at their first appearance. Mean numbers for eight microsatellite loci are shown. Vertical bars indicate *SE*s. Three time‐series data sets were used for this analysis (sessions 11–15 for grid A; sessions 2–6 and 11–15 for grid I). Null alleles were not considered

To investigate the timing of the appearance of new alleles, we counted alleles that had newly appeared in the first five sessions after a crash, in which each allele was counted only at the first appearance. Because two crash events occurred in grid I during the study period (i.e., sessions 1 and 10), three time‐series data sets were used for this analysis: sessions 11–15 for grid A and sessions 2–6 and 11–15 for grid I. This analysis revealed that more new alleles appeared during the early increasing phase immediately following a crash (Figure 8).

### 3.4 Temporal changes in cluster composition

**FIGURE 9:**
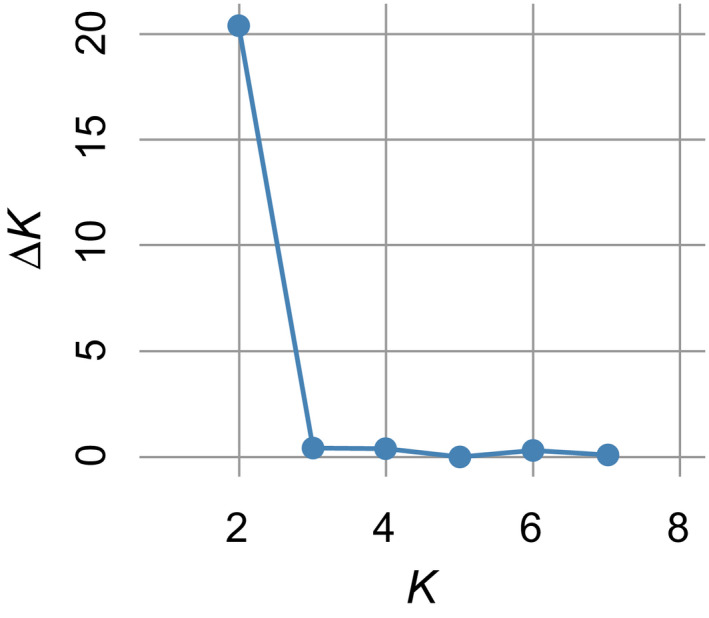
Δ*K* plot for all data from grids A and I, assessed using STRUCTURE. Genotype data at three microsatellite loci (MSCRB‐01, ‐04, and ‐13) were used. Other loci were excluded from the analysis due to the possible presence of null alleles

**FIGURE 10:**
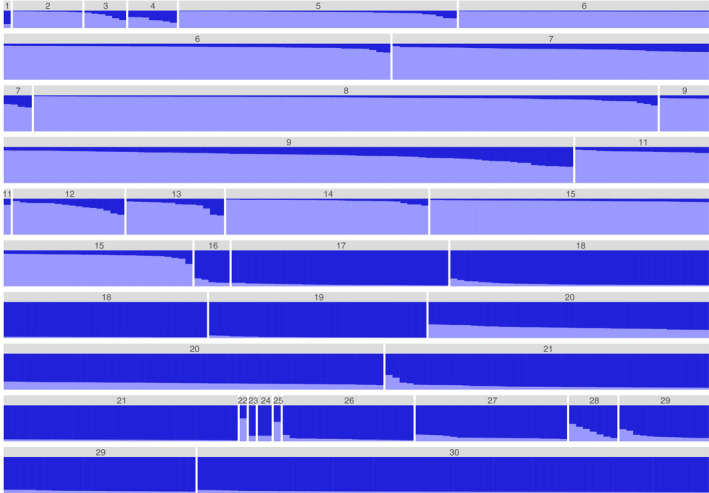
STRUCTURE plot for all data from grids A and I (*K* = 2). Numbers 1–15 and 16–30 above bars correspond to trapping sessions 1–15 at grid I and trapping sessions 1–15 at grid A, respectively. Genotype data for three microsatellite loci (MSCRB‐01, ‐04, and ‐13) were used. At grid I, no voles were captured in session 10 (May 2005)

First, STRUCTURE analyses were performed for all samples from both trapping grids, to analyze samples as if they had been collected at different sites. The highest Δ*K* value was observed at *K* = 2 (Figure 9). In the bar plot representing cluster assignment among individuals (hereafter referred to as a “STRUCTURE plot”), two clusters, each from one grid, were distinctly separated, although genotype data had only been used for three loci (MSCRB‐01, ‐04, and ‐13; Figure 10). Additionally, at each grid, temporal variability in cluster composition was relatively small among trapping sessions.

**FIGURE 11:**
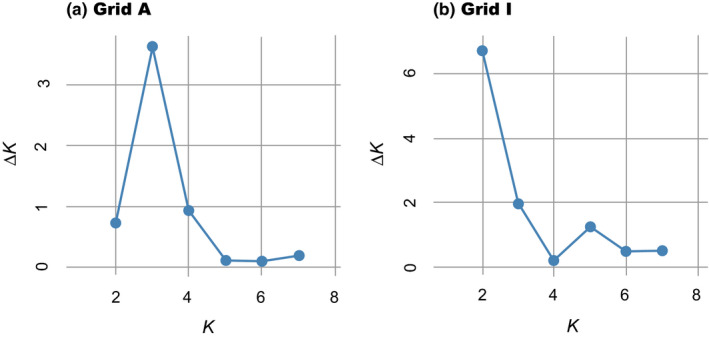
Δ*K* plots for (a) grid A, for which genotype data at three microsatellite loci (MSCRB‐01, ‐04, and ‐13) were used, and (b) grid I, for which genotype data at six microsatellite loci (MSCRB‐01, ‐04, ‐07, ‐09, ‐10, and ‐13) were used. For each grid, the loci used for analysis were limited due to the possible presence of null alleles at other loci

**FIGURE 12:**
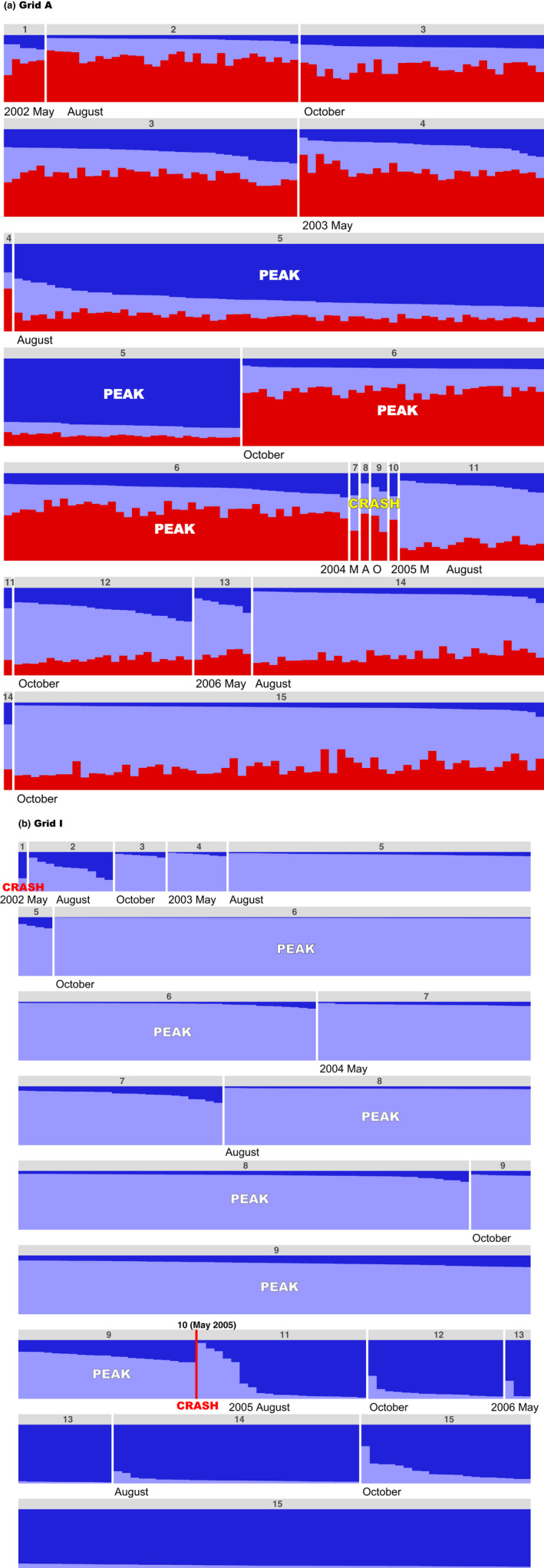
STRUCTURE plots for (a) grid A (*K* = 3), for which genotype data for three microsatellite loci (MSCRB‐01, ‐04, and ‐13) were used, and (b) grid I (*K* = 2), for which genotype data for six microsatellite loci (MSCRB‐01, ‐04, ‐07, ‐09, ‐10, and ‐13) were used. Numbers above bars are trapping session numbers. Density phases (i.e., crash and peak) were inferred. In grid I, no voles were captured in session 10 (May 2005)

When STRUCTURE analyses were performed separately for each trapping grid, the highest Δ*K* values were observed at *K* = 3 and *K* = 2 for grids A and I, respectively (Figure 11). At grid A, the proportions of three clusters varied considerably among individuals during the study period (Figure 12a). However, after the population crash (sessions 11–15), one previously minor cluster dominated, comprising a large proportion for each individual. At grid I, cluster compositions changed drastically before and after session 10, during which no voles were captured (Figure 12b). Relatively stable cluster compositions were observed during other trapping sessions; one cluster was predominant in sessions 2–9, while another dominated sessions 11–15.

**FIGURE 13:**
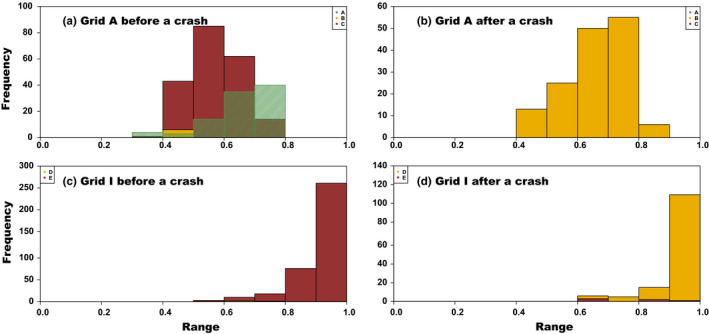
Changes in cluster composition before and after a crash. In each grid, *q* values corresponding to each individual's cluster assignment (its largest *q* value) were tallied before and after a crash. For grid A (*K* = 3: A, B, and C), (a) sessions 1–6 comprised the sessions before a crash, and (b) sessions 11–15 comprised those after a crash. For grid I (*K* = 2: D and E), (c) sessions 2–9 comprised the sessions before a crash, and (d) sessions 11–15 comprised those after a crash. Each cluster within each grid shares the same color

To clearly demonstrate temporal changes in cluster compositions, the frequency distributions of *q* values were compared before and after a crash in each grid, such that we used only the *q* values corresponding to each individual's cluster assignment, that is, its largest *q* value (Sacks et al., 2005). For grid A, before a crash (sessions 1–6), two of the three clusters accounted for higher proportions, and the frequency of the remaining cluster was very low (Figure 13a). By contrast, after a crash (sessions 11–15), all individuals in the latter cluster had the largest *q* values (Figure 13b). Similarly, for grid I, one of two clusters accounted for almost all of the *q* values before a crash (sessions 2–9) and after a crash (sessions 11–15), the other cluster accounted for a large amount (Figure 13c,d). Although similar critical changes in cluster composition occurred in both grids, the grids exhibited distinct frequency distribution patterns; throughout the study period, the largest class (0.9–1.0) of *q* values was most frequent at grid I, whereas values in the lower classes (0.5–0.8) were more frequent at grid A.

## 4 DISCUSSION

### 4.1 Resilience in genetic diversity

Our results revealed that genetic diversity was maintained even as the population sizes of gray‐sided voles fluctuated. Although levels of heterozygosity, allelic richness, and standardized individual heterozygosity were continuously high at both sites (Figures 4, 5, 6), allelic diversity was strongly, positively correlated with density (Figure 7). At each grid, if the density was similar, the mean number of alleles observed was also similar, irrespective of trapping sessions. Large fluctuations in density have been observed at both study sites since the late 1980s, and we suspect that such fluctuations have been happening for decades. Gauffre et al. (2014) found that, in the common vole *Microtus arvalis*, genetic diversity fluctuates at the local scale, while it remains constant at the regional scale. In gray‐sided vole populations, genetic diversity may be well‐preserved within metapopulations at the regional scale, whereas fluctuations in genetic diversity occur continuously at the local scale. Here, we observed that allele numbers were restored within a short period of time. The mutation rate for microsatellite loci is low (<7 × 10^−3^ per locus per gamete per generation; Brinkmann et al., 1998); thus, mutation cannot explain the observed accumulation of new alleles, because there were only a few generations of voles between periods of increasing and peak densities (Berthier et al., 2006). The rapid accumulation of new alleles after periods of low diversity must be due to individual dispersal. In the present study, significant departures from Hardy–Weinberg equilibrium were observed in some trapping sessions at both grids. The Hardy–Weinberg equilibrium may not be common in wild populations with high levels of admixture caused by dispersal (Waples & Gaggiotti, 2006). In addition to the presence of null alleles, active individual dispersal might have caused a slight shift from random to nonrandom mating at the study sites.

Gauffre et al. (2014) suggested that common voles travel over longer distances when density increases. Therefore, in the populations of gray‐sided voles studied here, individual dispersal must be the main contributor to the maintenance of genetic diversity. Although the number of alleles observed was correlated with density, this correlation was not linear; the rate of increase gradually declined with density (Figure 7). Furthermore, the alleles observed during the 5‐year study period did not always appear at the same time. These results imply that new genetic variants appear in populations mainly due to long‐distance dispersal during the early increasing phase when densities are very low; as density reaches high levels, the dispersal distance gradually decreases. Short‐distance dispersal appears to occur frequently under mid‐ to high‐density conditions, although little is known regarding individual dispersal behavior during transient and settlement stages in arvicoline rodents (Le Galliard et al., 2012). Our observation that more new alleles appeared during the early increasing phase supports this implication (Figure 8).

Notably, the relationship between density and allelic diversity was very similar between trapping grids (Figure 7). This implies that the two metapopulations represented by the trapping grids have similar levels of allelic diversity; moreover, individual dispersal acted in a similar manner to maintain genetic diversity within each metapopulation. From our human perspective, the trapping grids appeared to be located in habitats that are quite different, such that grid A was located in a shelterbelt and grid I was located in a low‐lying and damp area. However, these differences in habitat did not appear to affect vole dispersal behavior. Because we only surveyed genetic diversity at two sites, it remains unknown whether similar relationships can be found at other sites near the grids.

### 4.2 Dynamic changes in genetic composition

Because of moderate genetic differentiation among samples at each grid, STRUCTURE worked well for inferring the numbers of clusters (Latch et al., 2006). Our STRUCTURE analyses revealed that patterns of cluster composition differed between grids (Figure 12). At grid A, cluster composition varied largely among three clusters throughout the study period; one cluster dominated across individuals after the low‐density phase. In contrast, at grid I, cluster compositions among individuals remained relatively stable except for a sharp change before and after the low‐density phase. Furthermore, at grid I, based on the frequency distribution of *q* values among individuals, values in the largest class were most frequent (Figure 13), whereas lower‐class values were more frequent at grid A, despite both grids exhibiting a distinct change in cluster composition before and after a crash. The following factors may have contributed to the differences between grids.

Voles that have dispersed over long distances from remote sites may have cluster compositions that are quite different from those of the previous inhabitants. If there were no survivors in an area, the next generation would only comprise immigrants. At grid I, no voles were captured during trapping session 10 (Figure 3). During that session, we also did not capture any voles near the grid in our additional traps. Thus, at the low‐density phase, it is extremely unlikely that the voles captured originated from the area. It is more probable that the study site was populated again after the population crash by a few voles that had dispersed from distant areas; accordingly, their genetic compositions were different from those of the previous inhabitants. Conversely, at grid A and its surrounding area, voles were continuously captured even during the low‐density phase (sessions 7–10), although the numbers captured were very small. A substantial change in genetic composition (according to STRUCTURE analysis) might have occurred at grid I, but not at grid A, because of differences in the numbers of individuals that remained during periods of low density.

Furthermore, landscape features near the grids might have led to differences in cluster compositions between grids. For example, there was a paved road on a mound at the west side of grid I (Figure 2d). The road might have limited the immigration of voles with unique genetic compositions from the west side of the grid. Hence, at grid I, except for long‐distance dispersal during periods of extremely low densities, relatively stable genetic compositions might have been maintained during low‐ to high‐density phases at the western edge of the metapopulation. Conversely, grid A did not exhibit landscape features that might have limited individual dispersal; voles with various genetic compositions were likely able to emigrate into the grid from surrounding areas (Figure 2c). Accordingly, cluster compositions at this grid varied throughout the study period.

When the analysis was performed using all data from both grids, the cluster compositions exhibited considerable differences. Two clusters were detected (*K* = 2) and voles were sorted into distinct groups based on the grid in which they were captured, independent of trapping session (Figure 10). In the STRUCTURE plot, critical changes were not observed at low density for either grid; temporal variability in cluster composition was small among trapping sessions at each grid. Because there is an urbanized area between the two grids (Figure 2b), the rate of gene flow between grids may be low. Based on differences in the demographic, environmental, and historical processes that have led to the current genetic structure at each site, levels of genetic organization are also likely to differ between sites (Meirmans, 2015).

### 4.3 Genetic studies of cyclic lemming population density changes

Cyclic changes in population density have also been reported in arctic lemmings of two genera, *Dicrostonyx* and *Lemmus* (Ehrich et al., 2020). Although few genetic studies of lemmings have been conducted, they have similarly suggested the importance of migration (dispersal) in maintaining high genetic diversity in fluctuating populations. Ehrich et al. (2001) examined four microsatellite loci that were highly variable in all examined habitats of the North American collared lemming (*D*. *groenlandicus*) in the Canadian Arctic. Genetic differentiation was clearly observed among geographical regions but weaker among localities within regions, suggesting gene flow within regions. Lagerholm et al. (2017) reported that nine Norwegian lemming (*L. lemmus*) microsatellite loci exhibited high variability indicative of a weak population structure among Fennoscandian subregions and proposed that mass movements during periods of peak‐density act as pulses of gene flow between mountain tundra areas, thus helping to preserve genetic variation and counteract differentiation among subregions. However, these are snapshot studies of populations, and genetic diversity is not examined regularly in lemmings (Ehrich et al., 2020). Long‐term genetic studies are needed to address the role of density‐dependent dispersal in the maintenance of genetic diversity in cyclic lemming populations.

## 5 CONCLUSION

In this study, we found that density fluctuated at similar levels between the two study sites, although patterns of fluctuations were not synchronized. Allelic diversity was well‐preserved at each site, and the relationship between density and allelic diversity was similar between sites. In their review of population cycles, Norén and Angerbjörn (2014) concluded that the signatures of genetic drift and selection on population genetic diversity are weaker and obscured by density‐dependent dispersal. The present study adds to the growing body of evidence that dispersal usually overshadows the impact of other microevolutionary processes in cyclic populations.

Rikalainen et al. (2012) proposed that in addition to increased individual dispersal and the resulting accumulation of new alleles during peak phases, a constant presence and large effective population size facilitate the maintenance of high genetic diversity within fluctuating populations. In the gray‐sided vole, it is unknown how fluctuations in density affect metapopulations in which genetic diversity is totally preserved. Because a few voles were captured from local populations during low‐density phases, the effective population size of metapopulations at both study sites must be large. In addition to landscape features, which can affect connectivity among populations, fluctuations in density affect the spatial and temporal structure of metapopulations; genetic diversity is preserved within a metapopulation through density‐dependent individual dispersal. Further studies are needed to clarify how fluctuations in density influence metapopulations at the regional scale.

## CONFLICT OF INTEREST

None declared.

## AUTHOR CONTRIBUTION

**Yasuyuki Ishibashi:** Conceptualization (lead); Formal analysis (lead); Investigation (equal); Writing‐original draft (lead); Writing‐review & editing (lead). **Kenichi Takahashi:** Investigation (equal); Writing‐review & editing (supporting).

## Supporting information

Appendix S1

Appendix S2

Appendix S3

Appendix S4

Appendix S5

## Data Availability

Sampling locations and microsatellite genotypes were submitted to Dryad: https://doi.org/10.5061/dryad.mcvdncjzf.
