# Supplementary material for: Role of individual dispersal in genetic resilience in fluctuating populations of the gray‐sided vole Myodes rufocanus
Source: Ecol Evol. 2021 Feb 21;11(7):3407–21. doi: 10.1002/ece3.7300 (PMC8019057; doi:10.1002/ece3.7300)
Supplement: Supplementary file 1 — Appendix S1 [file ECE3-11-3407-s001.docx]

| **Appendix 1.** Genetic characteristics of eight microsatellite loci at each trapping session at grid A. Significant departure from Hardy-Weinberg equilibrium is indicated in red. N, sample size; Na, number of alleles; Ne, number of effective alleles; I, Shannon's information index; Ho, observed heterozygosity; He and uHe, expected and unbiased expected heterozygosity, respectively; F, fixation index. *Suspected null alleles identified using MICRO-CHECKER (Van Oosterhout et al., 2004) were not included in this number. | | | | | | | | | |
| --- | --- | --- | --- | --- | --- | --- | --- | --- | --- |
|  |  | MSCRB |  |  |  |  |  |  |  |
| Session # |  | 01 | 04 | 06 | 07 | 09 | 10 | 11 | 13 |
| 1 (May 2002) | N | 5 | 5 | 5 | 5 | 5 | 5 | 5 | 5 |
|  | Na | 7 | 5 | 9 | 7 | 5 | 7 | 6 | 5 |
|  | Ne | 6.250 | 4.545 | 8.333 | 5.556 | 3.125 | 6.250 | 4.167 | 3.846 |
|  | I | 1.887 | 1.557 | 2.164 | 1.834 | 1.359 | 1.887 | 1.609 | 1.471 |
|  | Ho | 1.000 | 0.600 | 1.000 | 1.000 | 0.800 | 1.000 | 1.000 | 0.600 |
|  | He | 0.840 | 0.780 | 0.880 | 0.820 | 0.680 | 0.840 | 0.760 | 0.740 |
|  | uHe | 0.933 | 0.867 | 0.978 | 0.911 | 0.756 | 0.933 | 0.844 | 0.822 |
|  | F | -0.190 | 0.231 | -0.136 | -0.220 | -0.176 | -0.190 | -0.316 | 0.189 |
| 2 (Aug 2002) | N | 31 | 31 | 31 | 31 | 31 | 31 | 31 | 31 |
|  | Na | 7 | 8 | 15* | 11 | 8 | 15 | 10 | 10 |
|  | Ne | 5.339 | 4.941 | 8.393 | 7.280 | 4.512 | 7.066 | 7.392 | 4.544 |
|  | I | 1.769 | 1.773 | 2.382 | 2.182 | 1.747 | 2.241 | 2.105 | 1.771 |
|  | Ho | 0.968 | 0.839 | 0.742 | 0.903 | 0.903 | 0.839 | 0.903 | 0.806 |
|  | He | 0.813 | 0.798 | 0.881 | 0.863 | 0.778 | 0.858 | 0.865 | 0.780 |
|  | uHe | 0.826 | 0.811 | 0.895 | 0.877 | 0.791 | 0.873 | 0.879 | 0.793 |
|  | F | -0.191 | -0.052 | 0.158 | -0.047 | -0.160 | 0.023 | -0.045 | -0.034 |
| 3 (Oct 2002) | N | 66 | 66 | 66 | 66 | 66 | 66 | 66 | 66 |
|  | Na | 11 | 9 | 13 | 14 | 11 | 17 | 13 | 11 |
|  | Ne | 5.577 | 6.415 | 9.957 | 5.739 | 4.942 | 7.377 | 7.993 | 6.345 |
|  | I | 1.934 | 2.011 | 2.400 | 2.035 | 1.879 | 2.303 | 2.229 | 2.021 |
|  | Ho | 0.848 | 0.909 | 0.818 | 0.773 | 0.788 | 0.818 | 0.879 | 0.803 |
|  | He | 0.821 | 0.844 | 0.900 | 0.826 | 0.798 | 0.864 | 0.875 | 0.842 |
|  | uHe | 0.827 | 0.851 | 0.906 | 0.832 | 0.804 | 0.871 | 0.882 | 0.849 |
|  | F | -0.034 | -0.077 | 0.090 | 0.064 | 0.012 | 0.054 | -0.004 | 0.047 |
| 4 (May 2003) | N | 31 | 31 | 31 | 31 | 31 | 31 | 31 | 31 |
|  | Na | 10 | 10 | 15 | 10* | 10 | 13 | 12 | 10 |
|  | Ne | 5.670 | 5.507 | 10.737 | 5.878 | 5.251 | 5.896 | 8.214 | 4.319 |
|  | I | 1.914 | 1.941 | 2.501 | 1.969 | 1.908 | 2.071 | 2.240 | 1.711 |
|  | Ho | 0.903 | 0.839 | 0.903 | 0.677 | 0.839 | 0.806 | 1.000 | 0.677 |
|  | He | 0.824 | 0.818 | 0.907 | 0.830 | 0.810 | 0.830 | 0.878 | 0.768 |
|  | uHe | 0.837 | 0.832 | 0.922 | 0.843 | 0.823 | 0.844 | 0.893 | 0.781 |
|  | F | -0.097 | -0.025 | 0.004 | 0.184 | -0.036 | 0.029 | -0.139 | 0.118 |
| 5 (Aug 2003) | N | 94 | 94 | 94 | 94 | 94 | 94 | 94 | 94 |
|  | Na | 11 | 10 | 18 | 14 | 12 | 15 | 15 | 11 |
|  | Ne | 5.605 | 5.930 | 11.672 | 5.806 | 7.400 | 6.944 | 9.272 | 5.899 |
|  | I | 1.949 | 2.009 | 2.562 | 1.966 | 2.204 | 2.186 | 2.376 | 1.967 |
|  | Ho | 0.787 | 0.904 | 0.894 | 0.755 | 0.862 | **0.915** | 0.926 | 0.904 |
|  | He | 0.822 | 0.831 | 0.914 | 0.828 | 0.865 | 0.856 | 0.892 | 0.830 |
|  | uHe | 0.826 | 0.836 | 0.919 | 0.832 | 0.869 | 0.861 | 0.897 | 0.835 |
|  | F | 0.042 | -0.088 | 0.023 | 0.088 | 0.004 | -0.069 | -0.037 | -0.089 |
| 6 (Oct 2003) | N | 80 | 80 | 80 | 80 | 80 | 80 | 80 | 80 |
|  | Na | 12 | 11 | 21 | 15 | 12* | 17 | 14 | 11 |
|  | Ne | 5.934 | 5.410 | 12.144 | 7.045 | 6.827 | 9.014 | 10.159 | 5.802 |
|  | I | 2.026 | 1.913 | 2.690 | 2.189 | 2.128 | 2.449 | 2.403 | 1.960 |
|  | Ho | 0.863 | 0.825 | 0.863 | 0.850 | 0.700 | 0.938 | 0.925 | 0.875 |
|  | He | 0.831 | 0.815 | 0.918 | 0.858 | 0.854 | 0.889 | 0.902 | 0.828 |
|  | uHe | 0.837 | 0.820 | 0.923 | 0.863 | 0.859 | 0.895 | 0.907 | 0.833 |
|  | F | -0.037 | -0.012 | 0.060 | 0.009 | 0.180 | -0.054 | -0.026 | -0.057 |
| 7 (May 2004) | N | 1 | 1 | 1 | 1 | 1 | 1 | 1 | 1 |
|  | Na | 2 | 2 | 2 | 2 | 2 | 2 | 2 | 2 |
|  | Ne | 2.000 | 2.000 | 2.000 | 2.000 | 2.000 | 2.000 | 2.000 | 2.000 |
|  | I | 0.693 | 0.693 | 0.693 | 0.693 | 0.693 | 0.693 | 0.693 | 0.693 |
|  | Ho | 1.000 | 1.000 | 1.000 | 1.000 | 1.000 | 1.000 | 1.000 | 1.000 |
|  | He | 0.500 | 0.500 | 0.500 | 0.500 | 0.500 | 0.500 | 0.500 | 0.500 |
|  | uHe | 1.000 | 1.000 | 1.000 | 1.000 | 1.000 | 1.000 | 1.000 | 1.000 |
|  | F | -1.000 | -1.000 | -1.000 | -1.000 | -1.000 | -1.000 | -1.000 | -1.000 |
| 8 (Aug 2004) | N | 1 | 1 | 1 | 1 | 1 | 1 | 1 | 1 |
|  | Na | 2 | 2 | 1 | 2 | 2 | 2 | 2 | 2 |
|  | Ne | 2.000 | 2.000 | 1.000 | 2.000 | 2.000 | 2.000 | 2.000 | 2.000 |
|  | I | 0.693 | 0.693 | 0.000 | 0.693 | 0.693 | 0.693 | 0.693 | 0.693 |
|  | Ho | 1.000 | 1.000 | 0.000 | 1.000 | 1.000 | 1.000 | 1.000 | 1.000 |
|  | He | 0.500 | 0.500 | 0.000 | 0.500 | 0.500 | 0.500 | 0.500 | 0.500 |
|  | uHe | 1.000 | 1.000 | 0.000 | 1.000 | 1.000 | 1.000 | 1.000 | 1.000 |
|  | F | -1.000 | -1.000 | #N/A | -1.000 | -1.000 | -1.000 | -1.000 | -1.000 |
| 9 (Oct 2004) | N | 2 | 2 | 2 | 2 | 2 | 2 | 2 | 2 |
|  | Na | 3 | 3 | 2 | 3 | 3 | 4 | 3 | 4 |
|  | Ne | 2.667 | 2.667 | 2.000 | 2.667 | 2.667 | 4.000 | 2.667 | 4.000 |
|  | I | 1.040 | 1.040 | 0.693 | 1.040 | 1.040 | 1.386 | 1.040 | 1.386 |
|  | Ho | 1.000 | 1.000 | 0.000 | 0.500 | 0.500 | 1.000 | 1.000 | 1.000 |
|  | He | 0.625 | 0.625 | 0.500 | 0.625 | 0.625 | 0.750 | 0.625 | 0.750 |
|  | uHe | 0.833 | 0.833 | 0.667 | 0.833 | 0.833 | 1.000 | 0.833 | 1.000 |
|  | F | -0.600 | -0.600 | 1.000 | 0.200 | 0.200 | -0.333 | -0.600 | -0.333 |
| 10 (May 2005) | N | 1 | 1 | 1 | 1 | 1 | 1 | 1 | 1 |
|  | Na | 2 | 2 | 2 | 1 | 2 | 2 | 2 | 2 |
|  | Ne | 2.000 | 2.000 | 2.000 | 1.000 | 2.000 | 2.000 | 2.000 | 2.000 |
|  | I | 0.693 | 0.693 | 0.693 | 0.000 | 0.693 | 0.693 | 0.693 | 0.693 |
|  | Ho | 1.000 | 1.000 | 1.000 | 0.000 | 1.000 | 1.000 | 1.000 | 1.000 |
|  | He | 0.500 | 0.500 | 0.500 | 0.000 | 0.500 | 0.500 | 0.500 | 0.500 |
|  | uHe | 1.000 | 1.000 | 1.000 | 0.000 | 1.000 | 1.000 | 1.000 | 1.000 |
|  | F | -1.000 | -1.000 | -1.000 | #N/A | -1.000 | -1.000 | -1.000 | -1.000 |
| 11 (Aug 2005) | N | 19 | 19 | 19 | 19 | 19 | 19 | 19 | 19 |
|  | Na | 6 | 6 | 13 | 7 | 11 | 7 | 9 | 8 |
|  | Ne | 4.658 | 2.798 | 8.299 | 4.878 | 7.443 | 3.967 | 5.967 | 5.157 |
|  | I | 1.658 | 1.337 | 2.322 | 1.722 | 2.162 | 1.619 | 1.969 | 1.820 |
|  | Ho | 1.000 | 0.789 | 0.895 | 0.789 | 0.842 | 0.895 | 0.842 | 0.842 |
|  | He | 0.785 | 0.643 | 0.880 | 0.795 | 0.866 | 0.748 | 0.832 | 0.806 |
|  | uHe | 0.807 | 0.660 | 0.903 | 0.817 | 0.889 | 0.768 | 0.855 | 0.828 |
|  | F | -0.273 | -0.228 | -0.017 | 0.007 | 0.027 | -0.196 | -0.012 | -0.045 |
| 12 (Oct 2005) | N | 22 | 22 | 22 | 22 | 22 | 22 | 22 | 22 |
|  | Na | 6 | 8 | 14* | 9 | 10 | 14* | 11 | 8 |
|  | Ne | 4.792 | 4.264 | 8.721 | 3.695 | 5.261 | 7.683 | 7.806 | 5.728 |
|  | I | 1.671 | 1.689 | 2.362 | 1.664 | 1.959 | 2.322 | 2.169 | 1.871 |
|  | Ho | 0.773 | 0.818 | **0.636** | 0.773 | 0.864 | **0.682** | 0.955 | 0.864 |
|  | He | 0.791 | 0.765 | 0.885 | 0.729 | 0.810 | 0.870 | 0.872 | 0.825 |
|  | uHe | 0.810 | 0.783 | 0.906 | 0.746 | 0.829 | 0.890 | 0.892 | 0.845 |
|  | F | 0.023 | -0.069 | 0.281 | -0.059 | -0.066 | 0.216 | -0.095 | -0.046 |
| 13 (May 2006) | N | 7 | 7 | 7 | 7 | 7 | 7 | 7 | 7 |
|  | Na | 7 | 7 | 10 | 5 | 7 | 7 | 8 | 6 |
|  | Ne | 6.125 | 5.765 | 8.909 | 3.630 | 5.765 | 6.125 | 6.533 | 4.261 |
|  | I | 1.871 | 1.834 | 2.243 | 1.438 | 1.847 | 1.871 | 1.970 | 1.593 |
|  | Ho | 0.857 | 0.714 | 1.000 | 0.714 | 0.857 | 1.000 | 1.000 | 0.714 |
|  | He | 0.837 | 0.827 | 0.888 | 0.724 | 0.827 | 0.837 | 0.847 | 0.765 |
|  | uHe | 0.901 | 0.890 | 0.956 | 0.780 | 0.890 | 0.901 | 0.912 | 0.824 |
|  | F | -0.024 | 0.136 | -0.126 | 0.014 | -0.037 | -0.195 | -0.181 | 0.067 |
| 14 (Aug 2006) | N | 37 | 37 | 37 | 37 | 37 | 37 | 37 | 37 |
|  | Na | 9 | 10 | 17* | 10 | 11 | 12 | 13 | 8 |
|  | Ne | 4.332 | 5.422 | 13.690 | 5.764 | 5.876 | 7.460 | 7.778 | 4.745 |
|  | I | 1.732 | 1.944 | 2.717 | 1.936 | 2.037 | 2.182 | 2.223 | 1.695 |
|  | Ho | 0.757 | 0.865 | **0.838** | 0.865 | 0.838 | 0.919 | 0.865 | 0.649 |
|  | He | 0.769 | 0.816 | 0.927 | 0.827 | 0.830 | 0.866 | 0.871 | 0.789 |
|  | uHe | 0.780 | 0.827 | 0.940 | 0.838 | 0.841 | 0.878 | 0.883 | 0.800 |
|  | F | 0.016 | -0.060 | 0.096 | -0.046 | -0.010 | -0.061 | 0.008 | 0.178 |
| 15 (Oct 2006) | N | 64 | 64 | 64 | 64 | 64 | 64 | 64 | 64 |
|  | Na | 9 | 10 | 23* | 13 | 11 | 13 | 12* | 10 |
|  | Ne | 5.389 | 5.638 | 14.198 | 6.435 | 6.169 | 9.362 | 8.368 | 5.945 |
|  | I | 1.868 | 1.964 | 2.820 | 2.114 | 2.066 | 2.391 | 2.276 | 1.947 |
|  | Ho | 0.719 | 0.781 | **0.750** | **0.797** | 0.891 | 0.828 | **0.719** | 0.813 |
|  | He | 0.814 | 0.823 | 0.930 | 0.845 | 0.838 | 0.893 | 0.880 | 0.832 |
|  | uHe | 0.821 | 0.829 | 0.937 | 0.851 | 0.844 | 0.900 | 0.887 | 0.838 |
|  | F | 0.118 | 0.050 | 0.193 | 0.057 | -0.063 | 0.073 | 0.184 | 0.023 |
