# Supplementary material for: Role of individual dispersal in genetic resilience in fluctuating populations of the gray‐sided vole Myodes rufocanus
Source: Ecol Evol. 2021 Feb 21;11(7):3407–21. doi: 10.1002/ece3.7300 (PMC8019057; doi:10.1002/ece3.7300)
Supplement: Supplementary file 2 — Appendix S2 [file ECE3-11-3407-s004.docx]

| **Appendix 2.** Genetic characteristics of eight microsatellite loci at each trapping session at grid I. Significant departure from Hardy-Weinberg equilibrium is indicated in red. N, sample size; Na, number of alleles; Ne, number of effective alleles; I, Shannon's information index; Ho, observed heterozygosity; He and uHe, expected and unbiased expected heterozygosity, respectively; F, fixation index. *Suspected null alleles identified using MICRO-CHECKER (Van Oosterhout et al., 2004) were not included in this number. †Data missing from a single individual. | | | | | | | | | |
| --- | --- | --- | --- | --- | --- | --- | --- | --- | --- |
|  |  | MSCRB |  |  |  |  |  |  |  |
| Session # |  | 01 | 04 | 06 | 07 | 09 | 10 | 11 | 13 |
| 1 (May 2002) | N | 1 | 1 | 1 | 1 | 1 | 1 | 1 | 1 |
|  | Na | 2 | 2 | 2 | 2 | 2 | 2 | 2 | 2 |
|  | Ne | 2.000 | 2.000 | 2.000 | 2.000 | 2.000 | 2.000 | 2.000 | 2.000 |
|  | I | 0.693 | 0.693 | 0.693 | 0.693 | 0.693 | 0.693 | 0.693 | 0.693 |
|  | Ho | 1.000 | 1.000 | 1.000 | 1.000 | 1.000 | 1.000 | 1.000 | 1.000 |
|  | He | 0.500 | 0.500 | 0.500 | 0.500 | 0.500 | 0.500 | 0.500 | 0.500 |
|  | uHe | 1.000 | 1.000 | 1.000 | 1.000 | 1.000 | 1.000 | 1.000 | 1.000 |
|  | F | -1.000 | -1.000 | -1.000 | -1.000 | -1.000 | -1.000 | -1.000 | -1.000 |
| 2 (Aug 2002) | N | 10 | 10 | 10 | 10 | 10 | 10 | 10 | 10 |
|  | Na | 7 | 7 | 10 | 7 | 8 | 9 | 6 | 6 |
|  | Ne | 5.263 | 5.263 | 5.714 | 4.000 | 6.250 | 4.878 | 3.175 | 5.128 |
|  | I | 1.782 | 1.782 | 2.037 | 1.609 | 1.934 | 1.851 | 1.429 | 1.697 |
|  | Ho | 0.800 | 0.800 | **0.600** | 0.700 | 1.000 | 0.700 | 0.800 | 0.800 |
|  | He | 0.810 | 0.810 | 0.825 | 0.750 | 0.840 | 0.795 | 0.685 | 0.805 |
|  | uHe | 0.853 | 0.853 | 0.868 | 0.789 | 0.884 | 0.837 | 0.721 | 0.847 |
|  | F | 0.012 | 0.012 | 0.273 | 0.067 | -0.190 | 0.119 | -0.168 | 0.006 |
| 3 (Oct 2002) | N | 6 | 6 | 6 | 6 | 6 | 6 | 6 | 6 |
|  | Na | 7 | 8 | 8 | 6 | 8 | 6 | 6 | 6 |
|  | Ne | 5.143 | 6.000 | 6.000 | 5.143 | 7.200 | 4.500 | 4.500 | 4.800 |
|  | I | 1.792 | 1.936 | 1.936 | 1.705 | 2.023 | 1.633 | 1.633 | 1.676 |
|  | Ho | 0.667 | 1.000 | 1.000 | 0.667 | 1.000 | 0.833 | 0.833 | 1.000 |
|  | He | 0.806 | 0.833 | 0.833 | 0.806 | 0.861 | 0.778 | 0.778 | 0.792 |
|  | uHe | 0.879 | 0.909 | 0.909 | 0.879 | 0.939 | 0.848 | 0.848 | 0.864 |
|  | F | 0.172 | -0.200 | -0.200 | 0.172 | -0.161 | -0.071 | -0.071 | -0.263 |
| 4 (May 2003) | N | 7 | 7 | 7 | 7 | 7 | 7 | 7 | 7 |
|  | Na | 4 | 5 | 10 | 6 | 7 | 6 | 6 | 6 |
|  | Ne | 3.161 | 3.920 | 7.000 | 4.900 | 4.900 | 4.455 | 4.455 | 4.667 |
|  | I | 1.240 | 1.470 | 2.144 | 1.673 | 1.767 | 1.611 | 1.631 | 1.649 |
|  | Ho | 0.857 | 1.000 | 0.857 | 1.000 | 1.000 | 0.714 | 0.857 | 0.857 |
|  | He | 0.684 | 0.745 | 0.857 | 0.796 | 0.796 | 0.776 | 0.776 | 0.786 |
|  | uHe | 0.736 | 0.802 | 0.923 | 0.857 | 0.857 | 0.835 | 0.835 | 0.846 |
|  | F | -0.254 | -0.342 | 0.000 | -0.256 | -0.256 | 0.079 | -0.105 | -0.091 |
| 5 (Aug 2003) | N | 40 | 40 | 40 | 40 | 40 | 40 | 40 | 40 |
|  | Na | 9 | 11 | 15 | 10 | 13 | 9 | 12 | 10 |
|  | Ne | 3.990 | 8.625 | 10.561 | 5.405 | 6.667 | 4.755 | 5.893 | 6.362 |
|  | I | 1.671 | 2.268 | 2.517 | 1.846 | 2.210 | 1.816 | 2.095 | 2.028 |
|  | Ho | 0.825 | 0.925 | 0.900 | 0.875 | 0.875 | 0.775 | 0.775 | 0.925 |
|  | He | 0.749 | 0.884 | 0.905 | 0.815 | 0.850 | 0.790 | 0.830 | 0.843 |
|  | uHe | 0.759 | 0.895 | 0.917 | 0.825 | 0.861 | 0.800 | 0.841 | 0.853 |
|  | F | -0.101 | -0.046 | 0.006 | -0.074 | -0.029 | 0.019 | 0.067 | -0.098 |
| 6 (Oct 2003) | N | 91 | 91 | 91 | 91 | 91 | 91 | 91 | 91 |
|  | Na | 10 | 13 | 23 | 11 | 12 | 13 | 12 | 12 |
|  | Ne | 4.875 | 7.831 | 11.438 | 6.173 | 7.757 | 5.757 | 6.182 | 6.673 |
|  | I | 1.864 | 2.203 | 2.712 | 1.981 | 2.227 | 2.067 | 2.137 | 2.082 |
|  | Ho | 0.824 | 0.824 | 0.967 | 0.835 | 0.901 | 0.835 | 0.857 | 0.857 |
|  | He | 0.795 | 0.872 | 0.913 | 0.838 | 0.871 | 0.826 | 0.838 | 0.850 |
|  | uHe | 0.799 | 0.877 | 0.918 | 0.843 | 0.876 | 0.831 | 0.843 | 0.855 |
|  | F | -0.037 | 0.055 | -0.060 | 0.003 | -0.034 | -0.011 | -0.023 | -0.008 |
| 7 (May 2004) | N | 49 | 49 | 49 | 49 | 49 | 49 | 49 | 49 |
|  | Na | 10 | 10 | 20* | 14 | 12 | 13 | 13 | 10 |
|  | Ne | 4.778 | 7.898 | 13.451 | 5.814 | 8.044 | 6.909 | 7.821 | 5.451 |
|  | I | 1.874 | 2.181 | 2.779 | 2.056 | 2.252 | 2.182 | 2.246 | 1.950 |
|  | Ho | 0.837 | 0.857 | 0.837 | 0.796 | 0.959 | 0.796 | 0.939 | 0.735 |
|  | He | 0.791 | 0.873 | 0.926 | 0.828 | 0.876 | 0.855 | 0.872 | 0.817 |
|  | uHe | 0.799 | 0.882 | 0.935 | 0.837 | 0.885 | 0.864 | 0.881 | 0.825 |
|  | F | -0.058 | 0.019 | 0.096 | 0.039 | -0.095 | 0.069 | -0.076 | 0.100 |
| 8 (Aug 2004) | N | 89 | 89 | 89 | 89 | 89 | 89 | 89 | 89 |
|  | Na | 10 | 11 | 22 | 13 | 12 | 14 | 12* | 11 |
|  | Ne | 6.028 | 7.909 | 12.445 | 5.016 | 8.791 | 6.670 | 8.440 | 6.282 |
|  | I | 2.010 | 2.175 | 2.775 | 1.932 | 2.277 | 2.159 | 2.303 | 2.069 |
|  | Ho | 0.831 | 0.876 | 0.921 | 0.809 | 0.854 | 0.876 | 0.798 | 0.888 |
|  | He | 0.834 | 0.874 | 0.920 | 0.801 | 0.886 | 0.850 | 0.882 | 0.841 |
|  | uHe | 0.839 | 0.878 | 0.925 | 0.805 | 0.891 | 0.855 | 0.886 | 0.846 |
|  | F | 0.003 | -0.003 | -0.002 | -0.010 | 0.036 | -0.031 | 0.095 | -0.056 |
| 9 (Oct 2004) | N | 88 | 88 | 88 | 88 | 88 | 88 | 88 | 88 |
|  | Na | 11 | 11 | 22 | 12 | 14 | 16 | 13 | 11 |
|  | Ne | 6.008 | 8.552 | 13.329 | 6.844 | 8.780 | 7.190 | 7.515 | 7.111 |
|  | I | 1.989 | 2.247 | 2.794 | 2.125 | 2.352 | 2.247 | 2.230 | 2.122 |
|  | Ho | 0.784 | 0.864 | 0.898 | 0.818 | 0.852 | **0.795** | 0.875 | 0.875 |
|  | He | 0.834 | 0.883 | 0.925 | 0.854 | 0.886 | 0.861 | 0.867 | 0.859 |
|  | uHe | 0.838 | 0.888 | 0.930 | 0.859 | 0.891 | 0.866 | 0.872 | 0.864 |
|  | F | 0.059 | 0.022 | 0.029 | 0.042 | 0.038 | 0.076 | -0.009 | -0.018 |
| 11 (Aug 2005) | N | 20 | 20 | 20 | 20 | 20 | 20 | 20 | 20 |
|  | Na | 6 | 8 | 13 | 9 | 11 | 8 | 10 | 7 |
|  | Ne | 2.312 | 5.096 | 8.511 | 4.969 | 5.839 | 3.239 | 6.452 | 4.396 |
|  | I | 1.172 | 1.785 | 2.315 | 1.827 | 2.032 | 1.533 | 2.041 | 1.657 |
|  | Ho | 0.600 | 0.950 | **0.950** | 0.800 | 0.950 | 0.750 | 0.950 | 0.800 |
|  | He | 0.568 | 0.804 | 0.883 | 0.799 | 0.829 | 0.691 | 0.845 | 0.773 |
|  | uHe | 0.582 | 0.824 | 0.905 | 0.819 | 0.850 | 0.709 | 0.867 | 0.792 |
|  | F | -0.057 | -0.182 | -0.076 | -0.002 | -0.146 | -0.085 | -0.124 | -0.036 |
| 12 (Oct 2005) | N | 16 | 16 | 16 | 16 | 16 | 16 | 16 | 16 |
|  | Na | 7 | 9 | 11 | 8 | 9 | 11 | 9 | 7 |
|  | Ne | 4.531 | 6.649 | 6.919 | 4.531 | 6.321 | 6.095 | 5.389 | 5.565 |
|  | I | 1.705 | 2.020 | 2.133 | 1.756 | 2.000 | 2.038 | 1.924 | 1.801 |
|  | Ho | 0.813 | 0.813 | 0.875 | 0.750 | 0.813 | 0.875 | 0.875 | 0.938 |
|  | He | 0.779 | 0.850 | 0.855 | 0.779 | 0.842 | 0.836 | 0.814 | 0.820 |
|  | uHe | 0.804 | 0.877 | 0.883 | 0.804 | 0.869 | 0.863 | 0.841 | 0.847 |
|  | F | -0.043 | 0.044 | -0.023 | 0.038 | 0.035 | -0.047 | -0.074 | -0.143 |
| 13 (May 2006) | N | 14 | 14 | 14 | 14 | 14 | 14 | 14 | 14 |
|  | Na | 8 | 8 | 11 | 7 | 9 | 7 | 9 | 3 |
|  | Ne | 5.939 | 5.681 | 8.522 | 4.404 | 6.644 | 4.558 | 7.127 | 2.405 |
|  | I | 1.898 | 1.878 | 2.246 | 1.638 | 2.026 | 1.659 | 2.046 | 0.953 |
|  | Ho | 1.000 | 0.929 | 1.000 | 0.929 | 0.857 | 0.857 | 1.000 | 0.714 |
|  | He | 0.832 | 0.824 | 0.883 | 0.773 | 0.849 | 0.781 | 0.860 | 0.584 |
|  | uHe | 0.862 | 0.854 | 0.915 | 0.802 | 0.881 | 0.810 | 0.892 | 0.606 |
|  | F | -0.202 | -0.127 | -0.133 | -0.201 | -0.009 | -0.098 | -0.163 | -0.223 |
| 14 (Aug 2006) | N | 29 | 29 | 29 | 29 | 29 | 29 | 28† | 29 |
|  | Na | 7 | 8 | 13 | 10 | 10 | 10 | 8 | 7 |
|  | Ne | 4.596 | 6.116 | 8.806 | 5.533 | 7.442 | 7.250 | 5.244 | 4.380 |
|  | I | 1.665 | 1.911 | 2.326 | 1.918 | 2.121 | 2.094 | 1.833 | 1.630 |
|  | Ho | 0.931 | 1.000 | 0.931 | **0.862** | 1.000 | 0.897 | 0.857 | 0.724 |
|  | He | 0.782 | 0.837 | 0.886 | 0.819 | 0.866 | 0.862 | 0.809 | 0.772 |
|  | uHe | 0.796 | 0.851 | 0.902 | 0.834 | 0.881 | 0.877 | 0.824 | 0.785 |
|  | F | -0.190 | -0.195 | -0.050 | -0.052 | -0.155 | -0.040 | -0.059 | 0.062 |
| 15 (Oct 2006) | N | 67 | 67 | 67 | 67 | 67 | 67 | 67 | 67 |
|  | Na | 8 | 11 | 23* | 10 | 13 | 12 | 14 | 11 |
|  | Ne | 5.044 | 7.814 | 11.111 | 5.363 | 9.920 | 6.864 | 7.596 | 4.845 |
|  | I | 1.740 | 2.159 | 2.694 | 1.876 | 2.428 | 2.152 | 2.222 | 1.810 |
|  | Ho | 0.791 | 0.821 | 0.746 | 0.866 | 0.910 | 0.881 | 0.925 | 0.761 |
|  | He | 0.802 | 0.872 | 0.910 | 0.814 | 0.899 | 0.854 | 0.868 | 0.794 |
|  | uHe | 0.808 | 0.879 | 0.917 | 0.820 | 0.906 | 0.861 | 0.875 | 0.800 |
|  | F | 0.013 | 0.059 | 0.180 | -0.064 | -0.013 | -0.031 | -0.066 | 0.041 |
