# Supplementary material for: Role of individual dispersal in genetic resilience in fluctuating populations of the gray‐sided vole Myodes rufocanus
Source: Ecol Evol. 2021 Feb 21;11(7):3407–21. doi: 10.1002/ece3.7300 (PMC8019057; doi:10.1002/ece3.7300)
Supplement: Supplementary file 3 — Appendix S3 [file ECE3-11-3407-s002.docx]

**Appendix 3.** Allelic richness and standardized individual heterozygosity at each trapping session. Allelic richness was calculated using the *hierfstat* package (Goudet, 2005) in R 3.6.2 software (R Development Core Team, 2019). Standardized individual heterozygosity is the proportion of heterozygous typed loci relative to the mean heterozygosity of typed loci (Coltman et al., 1999). N, sample size. SE, standard error.

**Allelic richness**

|  |  |  |  |  |  |  |  |  |  |  |  |  |
| --- | --- | --- | --- | --- | --- | --- | --- | --- | --- | --- | --- | --- |
| **Grid A** |  |  |  |  |  |  |  |  |  |  |  |  |
|  |  | MSCRB | |  |  |  |  |  |  |  |  |  |
| Trapping session# | N | 01 | 04 | 06 | 07 | 09 | 10 | 11 | 13 |  | Mean | SE |
| 1 | 5 | 1.933 | 1.867 | 1.978 | 1.911 | 1.756 | 1.933 | 1.844 | 1.822 |  | 1.881 | 0.026 |
| 2 | 31 | 1.826 | 1.811 | 1.895 | 1.877 | 1.791 | 1.873 | 1.879 | 1.793 |  | 1.843 | 0.015 |
| 3 | 66 | 1.827 | 1.851 | 1.906 | 1.832 | 1.804 | 1.871 | 1.882 | 1.849 |  | 1.853 | 0.012 |
| 4 | 31 | 1.837 | 1.832 | 1.922 | 1.843 | 1.823 | 1.844 | 1.893 | 1.781 |  | 1.847 | 0.015 |
| 5 | 94 | 1.826 | 1.836 | 1.919 | 1.832 | 1.869 | 1.861 | 1.897 | 1.835 |  | 1.859 | 0.012 |
| 6 | 80 | 1.837 | 1.820 | 1.923 | 1.863 | 1.859 | 1.895 | 1.907 | 1.833 |  | 1.867 | 0.013 |
| 7 | 1 | 2.000 | 2.000 | 2.000 | 2.000 | 2.000 | 2.000 | 2.000 | 2.000 |  | 2.000 | 0.000 |
| 8 | 1 | 2.000 | 2.000 | 1.000 | 2.000 | 2.000 | 2.000 | 2.000 | 2.000 |  | 1.875 | 0.125 |
| 9 | 2 | 1.833 | 1.833 | 1.667 | 1.833 | 1.833 | 2.000 | 1.833 | 2.000 |  | 1.854 | 0.038 |
| 10 | 1 | 2.000 | 2.000 | 2.000 | 1.000 | 2.000 | 2.000 | 2.000 | 2.000 |  | 1.875 | 0.125 |
| 11 | 19 | 1.807 | 1.660 | 1.903 | 1.817 | 1.889 | 1.768 | 1.855 | 1.828 |  | 1.816 | 0.027 |
| 12 | 22 | 1.810 | 1.783 | 1.906 | 1.746 | 1.829 | 1.890 | 1.892 | 1.845 |  | 1.838 | 0.020 |
| 13 | 7 | 1.901 | 1.890 | 1.956 | 1.780 | 1.890 | 1.901 | 1.912 | 1.824 |  | 1.882 | 0.019 |
| 14 | 37 | 1.780 | 1.827 | 1.940 | 1.838 | 1.841 | 1.878 | 1.883 | 1.800 |  | 1.848 | 0.018 |
| 15 | 64 | 1.821 | 1.829 | 1.937 | 1.851 | 1.844 | 1.900 | 1.887 | 1.838 |  | 1.864 | 0.014 |
|  |  |  |  |  |  |  |  |  |  |  |  |  |
| **Grid I** |  |  |  |  |  |  |  |  |  |  |  |  |
|  |  | MSCRB | |  |  |  |  |  |  |  |  |  |
| Trapping session# | N | 01 | 04 | 06 | 07 | 09 | 10 | 11 | 13 |  | Mean | SE |
| 1 | 1 | 2.000 | 2.000 | 2.000 | 2.000 | 2.000 | 2.000 | 2.000 | 2.000 |  | 2.000 | 0.000 |
| 2 | 10 | 1.853 | 1.853 | 1.868 | 1.789 | 1.884 | 1.837 | 1.721 | 1.847 |  | 1.832 | 0.019 |
| 3 | 6 | 1.879 | 1.909 | 1.909 | 1.879 | 1.939 | 1.848 | 1.848 | 1.864 |  | 1.884 | 0.011 |
| 4 | 7 | 1.736 | 1.802 | 1.923 | 1.857 | 1.857 | 1.835 | 1.835 | 1.846 |  | 1.837 | 0.019 |
| 5 | 40 | 1.759 | 1.895 | 1.917 | 1.825 | 1.861 | 1.800 | 1.841 | 1.853 |  | 1.844 | 0.018 |
| 6 | 91 | 1.799 | 1.877 | 1.918 | 1.843 | 1.876 | 1.831 | 1.843 | 1.855 |  | 1.855 | 0.013 |
| 7 | 49 | 1.799 | 1.882 | 1.935 | 1.837 | 1.885 | 1.864 | 1.881 | 1.825 |  | 1.863 | 0.015 |
| 8 | 89 | 1.839 | 1.878 | 1.925 | 1.805 | 1.891 | 1.855 | 1.886 | 1.846 |  | 1.866 | 0.013 |
| 9 | 88 | 1.838 | 1.888 | 1.930 | 1.859 | 1.891 | 1.866 | 1.872 | 1.864 |  | 1.876 | 0.010 |
| 10 | 0 | - | - | - | - | - | - | - | - |  | - | - |
| 11 | 20 | 1.582 | 1.824 | 1.905 | 1.819 | 1.850 | 1.709 | 1.867 | 1.792 |  | 1.794 | 0.036 |
| 12 | 16 | 1.804 | 1.877 | 1.883 | 1.804 | 1.869 | 1.863 | 1.841 | 1.847 |  | 1.849 | 0.011 |
| 13 | 14 | 1.862 | 1.854 | 1.915 | 1.802 | 1.881 | 1.810 | 1.892 | 1.606 |  | 1.828 | 0.035 |
| 14 | 29 | 1.796 | 1.851 | 1.902 | 1.834 | 1.881 | 1.877 | 1.824 | 1.785 |  | 1.844 | 0.015 |
| 15 | 67 | 1.808 | 1.879 | 1.917 | 1.820 | 1.906 | 1.861 | 1.875 | 1.800 |  | 1.858 | 0.016 |

**Standardized individual heterozygosity**

| **Grid A** |  |  |  |
| --- | --- | --- | --- |
| Trapping session# | N | Mean | SE |
| 1 | 5 | 1.043 | 0.082 |
| 2 | 31 | 1.029 | 0.025 |
| 3 | 66 | 0.989 | 0.018 |
| 4 | 31 | 0.990 | 0.029 |
| 5 | 94 | 1.035 | 0.016 |
| 6 | 80 | 1.019 | 0.017 |
| 7 | 1 | 1.192 | - |
| 8 | 1 | 1.043 | - |
| 9 | 2 | 0.894 | 0.149 |
| 10 | 1 | 1.043 | - |
| 11 | 19 | 1.028 | 0.039 |
| 12 | 22 | 0.948 | 0.042 |
| 13 | 7 | 1.022 | 0.051 |
| 14 | 37 | 0.983 | 0.023 |
| 15 | 64 | 0.939 | 0.021 |
|  |  |  |  |
|  |  |  |  |
| **Grid I** |  |  |  |
| Trapping session# | N | Mean | SE |
| 1 | 1 | 1.170 | - |
| 2 | 10 | 0.907 | 0.042 |
| 3 | 6 | 1.024 | 0.076 |
| 4 | 7 | 1.045 | 0.050 |
| 5 | 40 | 1.005 | 0.028 |
| 6 | 91 | 1.009 | 0.012 |
| 7 | 49 | 0.988 | 0.022 |
| 8 | 89 | 1.002 | 0.016 |
| 9 | 88 | 0.989 | 0.016 |
| 10 | 0 | - | - |
| 11 | 20 | 0.987 | 0.028 |
| 12 | 16 | 0.987 | 0.047 |
| 13 | 14 | 1.065 | 0.028 |
| 14 | 29 | 1.053 | 0.023 |
| 15 | 67 | 0.980 | 0.022 |
