# Supplementary material for: Role of individual dispersal in genetic resilience in fluctuating populations of the gray‐sided vole Myodes rufocanus
Source: Ecol Evol. 2021 Feb 21;11(7):3407–21. doi: 10.1002/ece3.7300 (PMC8019057; doi:10.1002/ece3.7300)
Supplement: Supplementary file 4 — Appendix S4 [file ECE3-11-3407-s003.docx]

**Appendix 4.** Test for linkage disequilibrium among eight microsatellite loci using the web version of Genepop 4.7.3 (Raymond & Rousset, 1995; Rousset, 2008; http://genepop.curtin.edu.au/). For each trapping grid (grids A and I), genotype data were analyzed for two trapping sessions, during which numbers of captured voles were relatively large and null alleles were likely absent, based on MICRO-CHECKER (Van Oosterhout et al., 2004) analysis. Only significant combinations are shown here (Bonferroni correction, *P* < 0.05/28).

--------------------------------------------------------------------------------------------------------------

**Grid A**

**For trapping session 3 (*N* = 66)**

**Locus#1 Locus#2 P-Value S.E.**

MSCRB04 MSCRB10 0.000000 0.000000

MSCRB07 MSCRB10 0.000000 0.000000

MSCRB09 MSCRB10 0.001690 0.001690

MSCRB10 MSCRB11 0.000000 0.000000

**For trapping session 5 (*N* = 94)**

**Locus#1 Locus#2 P-Value S.E.**

MSCRB01 MSCRB04 0.000000 0.000000

MSCRB07 MSCRB13 0.000000 0.000000

--------------------------------------------------------------------------------------------------------------

**Grid I**

**For trapping session 6 (*N* = 91)**

**Locus#1 Locus#2 P-Value S.E.**

MSCRB01 MSCRB06 0.000740 0.000740

MSCRB09 MSCRB10 0.000000 0.000000

MSCRB04 MSCRB11 0.000000 0.000000

MSCRB06 MSCRB11 0.000000 0.000000

MSCRB04 MSCRB13 0.000000 0.000000

**For trapping session 9 (*N* = 88)**

There were no significant combinations.

--------------------------------------------------------------------------------------------------------------
