# Supplementary material for: Role of individual dispersal in genetic resilience in fluctuating populations of the gray‐sided vole Myodes rufocanus
Source: Ecol Evol. 2021 Feb 21;11(7):3407–21. doi: 10.1002/ece3.7300 (PMC8019057; doi:10.1002/ece3.7300)
Supplement: Supplementary file 5 — Appendix S5 [file ECE3-11-3407-s005.docx]

**Appendix 5.** Evaluation of three models which describe the nonlinear relationship between density and allelic diversity.

# 1. Data

**Grid A Grid I**

N Mean N Mean

5 6.375 1 2.000

31 10.625 10 7.500

66 12.375 6 6.875

31 11.375 7 6.250

94 13.250 40 11.125

80 14.250 91 13.250

1 2.000 49 12.875

1 1.875 89 13.250

2 3.125 88 13.750

1 1.875 20 9.000

19 8.375 16 8.875

22 10.250 14 7.750

7 7.125 29 9.125

37 11.375 67 12.875

64 12.875

(N: no. of captured voles; Mean: mean no. of observed alleles for eight loci)

# 2. Three models evaluated by nls command in R

Model 1：Mean ~ alpha*N^beta

Model 2：Mean ~ SSasymp(N, a, b, c)

Model 3：Mean ~ a+b*log(N, base= exp(1))

# 3-1. Analysis for grid A

# (1) AIC and coefficient of determination R^2^ for the models

Model 1： AIC = 47.40851, R^2^ = 95.03146

Model 2： AIC = 43.80446, R^2^ = 96.57717

Model 3： AIC = 27.12957, R^2^ = 98.71322

# (2) Estimated parameters of the models

Model 1:

Estimate Std.Error tvalue Pr(>|t|)

alpha 3.15453 0.35984 8.766 8.10e-07***

beta 0.33986 0.02974 11.428 3.74e-08***

Model 2:

Estimate Std.Error tvalue Pr(>|t|)

a 13.1341 0.5056 25.978 6.46e-12***

b 1.8626 0.5032 3.702 0.00303 **

c -2.8256 0.1719 -16.438 1.36e-09***

Model 3:

Estimate Std.Error tvalue Pr(>|t|)

a 1.81392 0.25081 7.232 6.63e-06***

b 2.62684 0.08317 31.583 1.12e-13***

(Signif. codes: 0 ‘***’ 0.001 ‘**’)

# 3-2. Analysis for grid I

# (1) AIC and coefficient of determination R^2^ for the models

Model 1： AIC = 35.65153, R^2^ = 95.54578

Model 2： AIC = 40.36279, R^2^ = 94.59445

Model 3： AIC = 30.17382, R^2^ = 96.98802

# (2) Estimated parameters of the models

Model 1:

Estimate Std.Error tvalue Pr(>|t|)

alpha 3.59586 0.31163 11.54 7.47e-08 ***

beta 0.30002 0.02247 13.35 1.46e-08 ***

Model 2:

Estimate Std.Error tvalue Pr(>|t|)

a 13.4274 0.5337 25.158 4.50e-11 ***

b 3.0060 0.7629 3.941 0.00231 **

c -3.0912 0.1974 -15.657 7.25e-09 ***

Model 3:

Estimate Std.Error tvalue Pr(>|t|)

a 1.5599 0.4416 3.532 0.00413 **

b 2.6154 0.1331 19.657 1.71e-10 ***

(Signif. codes: 0 ‘***’ 0.001 ‘**’)
